# Stress Reduction Techniques for Health Care Providers Dealing With Severe Coronavirus Infections (SARS, MERS, and COVID-19): A Rapid Review

**DOI:** 10.3389/fpsyg.2020.589698

**Published:** 2020-12-10

**Authors:** Edward Callus, Barbara Bassola, Valentina Fiolo, Enrico G. Bertoldo, Silvana Pagliuca, Maura Lusignani

**Affiliations:** ^1^Clinical Psychology Service, IRCCS Policlinico San Donato, San Donato Milanese, Italy; ^2^Department of Biomedical Sciences for Health, Università degli Studi di Milano, Milan, Italy; ^3^Nursing School, ASST Grande Ospedale Metropolitano Niguarda, Milan, Italy

**Keywords:** COVID-19, coronavirus, health care workers, psychological intervention, stress reduction techniques, review

## Abstract

**Objective:**

A rapid review was conducted to identify the most effective stress reduction techniques for health care providers dealing with patients infected with severe coronavirus (SARS, MERS, and COVID-19).

**Methods:**

PubMed, PsychInfo, Embase, and CINAHL databases were searched to identify relevant studies. Searches were restricted by date (2000 until present). All empirical quantitative and qualitative studies in which relaxation techniques of various types implemented on health care providers caring for patients during severe coronavirus pandemics and articles that consider the implementation of mental health care services considered to be pertinent, such as commentaries, were included.

**Results:**

Fourteen studies met the selection criteria, most of which were recommendations. Only one study described a digital intervention, and user satisfaction was measured. In the recommendations, both organizational and individual self-care interventions were suggested.

**Conclusions:**

Further research is necessary to establish tailor-made effective stress reduction interventions for this population, during these challenging and particular times.

## Introduction

In the last 15 years, three global viral infectious diseases, severe acute respiratory syndrome (SARS), Middle East respiratory syndrome (MERS), and coronavirus disease (COVID-19), have occurred worldwide, putting human lives at risk and challenging the health care providers working in the frontline. The SARS pandemic was declared as “contained” by the World Health Organization (WHO) in 2003 (World Health Organization, 2003).

It was reported by the Centers of Disease Control and Prevention that a total of 8,096 people in 29 countries were infected by it, out of whom 774 of them died (Centers for Disease Control and Prevention, 2016).

After the SARS pandemic occurred, population studies have shown that these kinds of events can cause anxiety, depression, stress, sleep disorders, and post-traumatic stress disorder (PTSD) (Hawryluck et al., 2004; Wu et al., 2005). Specifically, it is an experience of uncontrollable and excessive concern related to a number of situations or activities. Symptomatology includes restlessness, fatigue, and difficulty in concentrating, irritability, muscle tension, and sleep disturbances. It is not surprising that the health care workers who find themselves working in this situation encounter a lot of stress linked to both their personal safety and the safety of their families; this is an addition to the burden of dealing with patients who can experience severe psychological distress.

Concerns from health care workers and psychological distress during the previous SARS outbreak were linked to increased work stress, social isolation, and health fears. Factors related to the psychological distress of health professionals were physical and emotional exhaustion due to an overloaded health system, rapidly changing medical information and procedures, media control, nursing care, perception of self-risk, lifestyle affected by the epidemic, and subjective vulnerability (Maunder, 2004; Wong et al., 2005; Styra et al., 2008). In addition, when faced with the possibility of having to work in a pandemic, a significant proportion of health care workers did not go to work, despite having a strong sense of duty (Ives et al., 2009; Martinese et al., 2009).

During the SARS pandemic, health care workers reported significantly higher levels of distress, including burnout psychological stress and post-traumatic stress, hostility, and somatization (Chen et al., 2005; Maunder et al., 2006). It was suggested that healthcare workers should be screened when it comes to psychological distress, in order to provide psychological support (Chen et al., 2005). Prevention programs during SARS have been effective in diminishing anxiety and depression and improving sleep and quality of life (Chen et al., 2006).

In a cross-sectional study of 1,257 health care workers in 34 hospitals equipped with fever clinics or wards for patients with COVID-19 in multiple regions of China, a considerable proportion of health care workers reported experiencing symptoms of depression, anxiety, insomnia, and distress. It was suggested that, among Chinese health care workers exposed to COVID-19, women, nurses, those in Wuhan, and frontline health care workers had a high risk of developing unfavorable mental health outcomes and may need psychological support or interventions (Lai et al., 2020).

Among the health care providers, the frontline workers involved directly in handling COVID-19 patients are exposed at greater risk than others. Many reasons were found in adverse psychological outcomes ranging from excessive workload/work hours to inadequate personal protective equipment, over-enthusiastic media news, and feeling inadequately supported (Spoorthy et al., 2020).

Pervasive psychological problems have appeared among health care workers during the COVID-19 pandemic. The prevalence of symptoms of anxiety, depression, insomnia, and the overall psychological problems in health care workers during the COVID-19 pandemic in China was 46.04, 44.37, 28.75, and 56.59%, respectively (Que et al., 2020).

It has been demonstrated in various studies that relaxation techniques, such as the progressive muscular relaxation of Jacobson, the Mindfulness Based Stress Reduction techniques, and the relaxation techniques described by Benson, reduce stress, anxiety, and depression and improve quality of life in both patients and health care workers (Zinn, 1990; Botha et al., 2015; Carver and O’Malley, 2015; Greenlee et al., 2017; Tsitsi et al., 2017; Harorani et al., 2019; Ibrahim et al., 2019).

However, medical and nursing staff may be unable or reluctant to participate in psychological initiatives at the time of crisis (Chen et al., 2020). Initiatives such as staff training on psychological aspects in patient management and training in relaxation techniques (Chen et al., 2020) could therefore be useful, although the best approach during this pandemic is still unknown (Chen et al., 2020; Kang et al., 2020a; Xiang et al., 2020).

It is especially important to take into consideration the psychological care needs treatment preferences of the health care workers facing these kinds of situations. In one study, the impact on mental health care and perceptions of psychological care of the medical and nursing staff was explored in Wuhan (Kang et al., 2020b). The health care workers were divided into clusters according to their mental health disturbance: mild, moderate, and severe. When it comes to the interest in psychological care, the ones with subthreshold disturbances most wanted to obtain skills to help alleviate others’ psychological distress, whereas other medical and nursing staff most wanted to obtain self-help skills.

In the cluster where higher levels of mental health problems were reported, the medical and nursing staff showed more interest in skills for self-rescue and showed more urgent desires to seek help from psychotherapists and psychiatrists. Medical and nursing staff with subthreshold disturbances did not think they needed help from others. The other workers saw a greater need to obtain help from professionals than from close family and friends. Also, the modalities of obtaining services vary according to their levels of mental health problems. Medical and nursing staff with subthreshold and mild disturbances preferred to obtain such services from media sources, while staff with heavier burdens wanted to seek services directly from professionals (Kang et al., 2020b).

For this reason, it is important to identify what kind of interventions could be most effective in this population, also when it comes to self-help materials such as relaxation recordings, which can be delivered also without the health care workers having direct contact with the professionals.

## Methodology

This rapid review was aimed to identify the most effective stress reduction techniques for health care providers dealing with patients infected with severe coronavirus (SARS, MERS, and COVID-19).

In particular, there was a focus on the best practices and interventions that aimed at reducing psychological distress among health care professionals dealing with patients infected with severe coronavirus infections. Additionally, the delivery mechanisms of the identified interventions, the instruments used to test their efficacy, the determinants of their effectiveness, and their impact on specific psychological variables were investigated.

It was decided to focus only on severe coronaviruses because they have similar characteristics, and the objective of the review was to identify interventions that were specific to the health care providers who were facing similar situations.

The main question this rapid review aims to answer is:

• Which are the most effective stress reduction techniques for health care providers dealing with patients infected with severe coronavirus (SARS, MERS, and COVID-19)?

The secondary questions that we explored are:

• Which is the most effective manner of delivery considering the severe coronavirus characteristics?
• What instruments are utilized to measure stress reduction techniques’ efficacy?
• Which psychological variables are affected by stress reduction techniques?
• Which factors influence the effectiveness of stress reduction techniques’ application?

PubMed, PsychInfo, Embase, and CINAHL databases were searched to identify relevant studies. Searches were restricted by date (2000 until present). Searches were conducted by two authors independently between 3rd and 18th June 2020.

The keywords utilized in the various databases can be seen in the Supplementary Material (database search strings).

All studies and any systematic reviews identified during the screening process were reference checked to identify additional studies. During the preliminary screening of literature, two authors worked independently. Google Scholar was searched as other source.

The objectives were formulated according to the Cochrane Systematic Review indications (Thomas et al., 2019) and following the Cochrane indications and training materials for rapid reviews.

The eligibility criteria were referable to the type of participants; the studies had to concern health professionals, even if not exclusively; they had to indicate a technique, model, or recommendations for stress reduction; they had to relate to a problem or variable of psychological distress in health professionals during outbreaks of SARS, MERS, or COVID-19, and mental health outcomes.

All empirical quantitative and qualitative studies in which relaxation techniques of various types implemented on health care providers caring for patients during severe coronavirus pandemics and articles that consider the implementation of mental health care services considered to be pertinent, such as commentaries, were included. In addition, there was an exploration of any indicators of effectiveness in lowering psychological distress including anxiety, depression, PTSD, burnout, and others. Studies relating to the stress conditions of operators not related to epidemics and which did not propose interventions or recommendations were excluded. Information extracted from studies and reviewed included psychological distress variables, intervention, efficacy measurements, follow-up results, and country of study.

One reviewer screened all titles and abstracts (BB), considering the focus of the review and the inclusion and exclusion criteria. In the presence of uncertainties, the full text of the article was consulted. After that, two separate reviewers identified the definitive list by consulting the full texts of all the articles (EGB and EC). The quality evaluation of included literature was performed using the AGREE II-Global Rating Scale (AGREE II-GRS) Instrument (Brouwers et al., 2016); the GRADE approach was used to interpret the results (Langendam et al., 2013) and to create Table 1 (“Quality evaluation of the studies”).

**TABLE 1:** Quality evaluation of the studies.

**Authors and year**	**Rate the overall quality of the guideline development methods Lowest quality (1) Highest quality (7**)	**Rate the overall quality of the guideline presentation Lowest quality (1) Highest quality (7)**	**Rate the completeness of reporting Lowest quality (1) Highest quality (7)**	**Rate the overall quality of the guideline recommendations Lowest quality (1) Highest quality (7)**	**Rate the overall quality of the guideline Lowest quality (1) Highest quality (7)**	**I would recommend this guideline for use in practice Lowest quality (1) Highest quality (7)**	**I would make use of a guideline of this quality in my professional decisions Lowest quality (1) Highest quality (7)**
Blake et al., 2020	NP	NP	NP	NP	NP	NP	NP
De Mei et al., 2020	3	4	2	7	4	5	3
Fessell and Cherniss, 2020	2	5	2	7	4	5	3
Galbraith et al., 2020	6	6	2	7	6	6	5
Hedderman et al., 2020	3	5	3	7	5	5	4
Kar et al., 2020	4	6	2	7	5	6	5
Maben and Bridges, 2020	4	6	3	7	6	6	5
Moazzami et al., 2020	NP	NP	NP	NP	NP	NP	NP
Mukhtar, 2020	2	4	3	7	4	5	3
Petzold et al., 2020	5	7	5	7	6	7	6
Sasangohar et al., 2020	5	6	5	7	6	6	6
Shanafelt et al., 2020	6	5	5	7	5	6	5
Sultana et al., 2020	5	4	5	7	5	6	5
World Health Organization, 2020	4	3	4	7	5	6	5

**NP, not pertinent*.*

## Results

**FIGURE 1:**
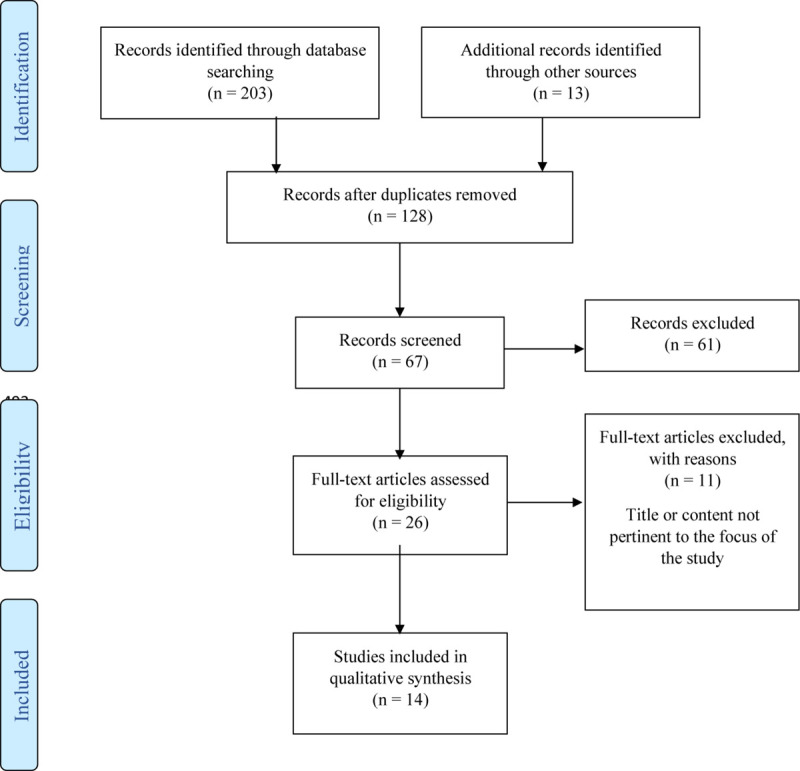
Flow diagram of the selected studies.

The workflow of the article selections can be seen in Figure 1, and the pertinent articles are reported in Table 2.

**TABLE 2:** Summary of the selected studies.

**Authors, year, and country**	**Title**	**Psychological distress variables**	**Intervention**	**Efficacy measurements**	**Follow-up results**
Blake et al., 2020—United Kingdom	Mitigating the Psychological Impact of COVID-19 on Healthcare Workers: A Digital Learning Package	Rest, work breaks, sleep, shift work, fatigue, healthy lifestyle behaviors, moral injury, coping, guilt, grief, fear, anxiety, depression, preventing burnout, and psychological trauma	The implementation of an e-package including evidence-based guidance, support, and signposting relating to psychological well-being for all United Kingdom health care employees	The e-package reported high user satisfaction with content, usability, and utility. All of the pre-defined success criteria were met for the fidelity assessment and implementation qualities.	Within just 7 days of release, 82% of participants reported having used the information provided in their work or home lives, and 100% would use it in the future.
De Mei et al., 2020—Italy	COVID-19: stress management among healthcare workers	Feeling angry, hostile, frustrated or helpless, depression, anxiety, insomnia, and increasing consumption of caffeine and tobacco	Advice for health care workers	Not tested	NA
Fessell and Cherniss, 2020—United States	Coronavirus Disease 2019 (COVID-19) and Beyond: Micro practices for Burnout Prevention and Emotional Wellness	Burnout	Recommendation about: mindfulness micro practice and self-reported stress levels via the widely used Depression Anxiety Stress Scales-21	Not tested	NA
Galbraith et al., 2020—United Kingdom	The mental health of doctors during the COVID-19 pandemic	Stress, stigma, suicidal ideation, feelings of shame, and professional failure	Recommendations about: managing doctors’ stress at the organizational level (PPE, peer support training) and stress management at the individual level (mindfulness)	Not tested	NA
Hedderman et al., 2020—Ireland	Mindfulness moments for clinicians in the midst of a pandemic	Burnout, distress, emotional suffering, fear of contamination for themselves and loved ones, the breakdown of social support systems, the deaths of colleagues, and increased psychological distress	Tips for mindfulness moments for clinicians (MMFC) (mindfulness-based cognitive therapy (MBCT) programs; the RAIN (Recognize, Allow, Investigate, and Nurture) practice	Not tested	NA
Kar et al., 2020—India, Bangladesh, United Kingdom, and Nepal	Coping with Mental Health Challenges During COVID-19	Physical exhaustion, fear, emotional disturbance, sleep disorders, depressive symptoms, anxiety, suicidality, PTSD, and burnout	Recommendations for effective coping with mental health challenges: adequate awareness about the COVID-19; preparedness to meet the challenges; ignoring fake news and social media posts; regular scheduling of the daily activities; recreational activities and relaxation exercises; approaching health care system; positive thinking and installation of hope	Not tested	NA
Maben and Bridges, 2020—United Kingdom	Covid-19: Supporting nurses’ psychological and mental health	Levels of occupational stress and resulting distress, concern for personal or family health, concern with the ethical obligations of continuing to provide care, concerns about shortages of staff and of personal protective equipment	Evidence-based psychological support: self-support (adequate food, shelter, rest, sleep and safety, calming strategies), peer support (peer support conversation), team support (buddying with more experienced colleagues), manager, and leader	Not tested	NA
Moazzami et al., 2020—Iran	COVID-19 and telemedicine: Immediate action required for maintaining healthcare providers well-being	Long work hours, sleep disturbances, debilitating fatigue, and the risk of getting infection and put their family at risk of a life-threatening condition	Recommendation of telemedicine	Not tested	NA
Mukhtar, 2020—Pakistan	Mental health and emotional impact of COVID-19: Applying Health Belief Model for medical staff to general public of Pakistan	Anxiety of falling sick or fear of death, sense of helplessness, hopelessness, exhaustion and burnout, nervous anticipation negative emotions, work–life balance	Recommendation about: Health Belief Model (HBM)	Not tested	NA
Petzold et al., 2020—Germany	Dealing with Psychological Distress by Healthcare Professionals During the COVID-19 Pandemia		The recommendations of the World Health Organization, the United Nations and the International Red Cross Society are summarized in the article	Not tested	NA
Sasangohar et al., 2020—United States	Provider burnout and fatigue during the COVID-19 pandemic: lessons learned from a high-volume intensive care	Occupational fatigue and burnout	Recommendations and policy implication at National, Regional and Organizational level	Not tested	NA
Shanafelt et al., 2020—United States	Understanding and Addressing Sources of Anxiety Among Health Care Professionals During the COVID-19 Pandemic	Sources of Anxiety: personal protective equipment, personal and family exposure to COVID-19, testing access, uncertainty about organization support, school closures, personal and family needs, being able to provide competent care, access to up-to-date information	Key components of how organization can respond	Not tested	NA
Sultana et al., 2020—Bangladesh	Burnout Among Healthcare Providers During COVID-19 Pandemic: Challenges and Evidence-based Interventions	Burnout, sleep deprivation, depression, and suicidal thoughts	Strategies recommended for addressing burnout among health care providers: provide awareness, mindfulness and self-care practices, availability of mental health services, digital technologies, organization-directed interventions	Not tested	NA
World Health Organization, 2020 —Switzerland	Healthcare Personnel and First Responders: How to Cope with Stress and Build Resilience During the COVID-19 Pandemic	Stress, anxiety, and fear	Tips to cope and enhance resilience	Not tested	NA

**NA, not available*.*

The search for documents produced 67 records. Forty-one of them were excluded because they were considered irrelevant after an independent analysis of title and abstract. The remaining 26 were analyzed in full text independently among the authors to assess their adherence to the selection criteria and 16 were excluded. Fourteen met the selection criteria and, after quality appraisal, were included in the narrative review (see Table 2 for the selected studies).

One of the selected studies (Blake et al., 2020) is an intervention study, 1 is a case study (Sasangohar et al., 2020), 11 are opinions and recommendations (De Mei et al., 2020; Fessell and Cherniss, 2020; Galbraith et al., 2020; Hedderman et al., 2020; Kar et al., 2020; Maben and Bridges, 2020; Moazzami et al., 2020; Petzold et al., 2020; Shanafelt et al., 2020; Sultana et al., 2020; World Health Organization, 2020), and 1 is a letter to editor (Mukhtar, 2020). The quality of the studies was moderate to high. As reported by some of the authors, few of the reviewed considerations, recommendations, and suggestions have substantial evidence to support them. Some are based on direct requests from health care professionals and experience. All studies are written in English, except Petzold et al. (2020), which is written in German with abstracts in both languages, and De Mei et al. (2020), which is available in both English and Italian.

The purposes of the reviewed studies were to suggest interventions or recommendations and specific policy organizational recommendations, for the reduction of stress and psychological burden in health professionals during COVID-19 (De Mei et al., 2020; Fessell and Cherniss, 2020; Galbraith et al., 2020; Hedderman et al., 2020; Kar et al., 2020; Maben and Bridges, 2020; Moazzami et al., 2020; Petzold et al., 2020; Shanafelt et al., 2020; Sultana et al., 2020; World Health Organization, 2020) and to propose strategies and interventions such as mindfulness (Hedderman et al., 2020), telemedicine (Moazzami et al., 2020), and health model (Mukhtar, 2020). There are no differences between the recommendations referred to different populations and cultures. All studies are aimed at health professionals (De Mei et al., 2020; Petzold et al., 2020; Shanafelt et al., 2020; Sultana et al., 2020; World Health Organization, 2020), some at specific figures such as doctors (Fessell and Cherniss, 2020; Galbraith et al., 2020) or nurses (Maben and Bridges, 2020). Kar et al. (2020) include indications for the general population. None of the studies found discussed or proposed interventions or recommendations addressed to health care professionals involved in the SARS and MERS epidemics (Liu et al., 2020).

## Relaxation Strategies During Pandemics

The study “Effects of progressive muscle relaxation on anxiety and sleep quality in patients with COVID-19” was conducted on 51 patients who tested positive for COVID-19 and admitted to the general hospital of Hainan from January 01. As of February 16, 2020, progressive muscle relaxation (PRM) was seen to have a positive effect on improving sleep quality and reducing anxiety in COVID-19 patients. The cause of the decrease in anxiety in patients after PMR practice could be the balance between the anterior nucleus and the hypothalamic nucleus. In fact, it has been shown that by reducing the activity of the sympathetic nervous system, it is possible to prevent the side effects of stress and anxiety and increase physical and mental relaxation (Ferendiuk et al., 2019).

In an attempt to develop a clinical protocol aimed at reducing the negative effects of the current pandemic on the psychophysical health of health care personnel and patients, further characteristics of relaxation practices were evaluated and investigated.

Health care personnel are subjected to numerous stressful factors during sometimes prolonged work shifts: taking care of more or less serious COVID-19-positive patients, the worry of a possible infection from COVID-19, the fear of being able to infect loved ones, quickly adapting to the reorganization of Operational Units and consequently to quickly changing their duties, working alongside unknown colleagues or doctors from an operational point of view, sustaining much heavier work rates, etc. All these factors lead to the accumulation of stress, which, over time, risks becoming chronic. This condition leads to an inevitable relapse on both a psychological and physiological level such as “chest and non-diaphragmatic breathing, muscle tension more intense than normal, a rapid heartbeat, a condition of sympathetic tone, the appearance of negative fantasies or a chronic fear” (Rispoli, 1999).

Breathing, for example, has been evaluated as a fundamental element for the mindful meditation practices introduced by Kabat-Zinn, as it allows you to pay “non-judgmental attention to your cognitive, emotional and physical experiences, while reorienting your concentration. on respiratory sensations to promote cognitive and emotional regulation and progressively relaxed districts” (Azam et al., 2019).

The main findings of the 2011 “Targeting the restricted α-subunit repertoire of airway smooth muscle GABAA receptors augments airway smooth muscle relaxation” study refer to the fact that human airway smooth muscle possesses GABAA receptors (gamma-aminobutyric acid) with a limited (but conserved) α-subunit phenotype that can be pharmacologically targeted by selective agonists to generate electrophysiological changes and facilitate relaxation of pre-contracted smooth muscle. The activation of the receptor can directly and spontaneously relax the pre-contracted smooth muscle of the airways by a variety of procontractile agents (Gallos et al., 2012).

Furthermore, in a review of the scientific literature (Pavan and Palese, 2016), it was shown that GABA is one of the most important inhibitory neurotransmitters and influences mood and emotions. Low levels are associated with depression and sleep disturbances, conditions also found in people with PTSD, as well as in anxiety situations in which there is a reduction in GABA (Rispoli, 2016).

The scientific evidences cited so far show the importance of increasing the presence of GABA neurotransmitters in the body especially in this moment of COVID-19 emergency, both for patients with respiratory problems and for all those, including patients, doctors, health workers, and the civilian population, who experience a strong state of anxiety.

Another study highlighted the beneficial effects of relaxation techniques in asthmatic patients by measuring lung function. The results show that the use of relaxation as an auxiliary treatment appears to help asthma patients better manage stress and prevent further attacks, thus improving their quality of life (Pourdowlat et al., 2019). Lahmann et al. (2009) had previously obtained similar results by evaluating the impact of relaxation and guided imagery techniques on asthmatic patients.

A recent review of the literature (Tarsha et al., 2020) found that body-oriented psychological therapies were effective in reducing headaches caused by a state of tension, non-cardiac (non-specific) chest pain, psychosomatically influenced asthma diseases, and irritable bowel disease as demonstrated in other studies (Loew et al., 2001; Lahmann et al., 2008a,b, 2009). The importance of the body in psychological therapies has already been highlighted for some time by Rispoli (1999, 2004, 2016) who already in 1999 spoke about the unity of body and mind, overcoming this dichotomy. His theoretical approach focused on targeted interventions on the body to alleviate psychological pathologies, strictly connected to the physical and physiological level, and fits into this perspective of body and mind integration (Rispoli, 2016).

## Interventions

In their study, Blake et al. (2020) describe the development and evaluation of a digital package using the Agile methodology, in the United Kingdom. The study includes three phases: a content development phase, a peer review phase, and a package implementation and evaluation phase. The package includes an evidence-based guide to support the psychological well-being for all United Kingdom health workers. The package outlines the actions that team leaders can undertake to provide psychologically safe spaces for workers, to provide a guide to reduce social stigma, and to increase peer and family support, self-care strategies related to sleep and rest, shift work, fatigue, and healthy lifestyle behaviors; it also includes emotion management strategies such as guilt, pain, fear, anxiety, depression, burnout prevention, and psychological trauma.

### Enhancing Awareness

The revised studies report to inform health care personnel about the professional stress risks associated with the emergency care. Awareness can reduce stigma to mental health conditions such as burnout and develop resilience in the health care provider by preventing burnout (Sultana et al., 2020). Adequate awareness of COVID-19 and regular updates about appropriate precautionary measures are recommended (Kar et al., 2020).

### Self-Care Interventions

Positive mental health can prevent work-related stress and burnout and should be promoted among health professionals in COVID-19. Several strategies are recommended for reducing the workload, such as mindfulness and promoting self-care (Sultana et al., 2020).

Mindfulness training is recommended for health care professionals because it can promote self-care and well-being (Fessell and Cherniss, 2020; Sultana et al., 2020; World Health Organization, 2020). Mindfulness programs are also recommended because they can increase resilience to stress, quality of professional life, and self-compassion.

Compassion for both self and others in a clinical setting is a necessary component to facilitate a therapeutic environment. Self-compassionate people react to adversaries’ events in a more emotionally regulated model. Self-compassion is associated with a series of psychological strengths such as resilience, happiness, optimism, wisdom, curiosity, courage, exploration, and emotional intelligence (Hedderman et al., 2020).

Mindfulness-based interventions are particularly suitable for high-stress work contexts, can be practiced privately or in groups, in almost all environments, and can be conducted as briefly as possible (Galbraith et al., 2020). Mindfulness is recommended also for reducing work stress and suicidal ideation (Galbraith et al., 2020).

The promotion of self-care must start from the response to the essential needs of drinks, food, rest, and sleep (Maben and Bridges, 2020; Petzold et al., 2020; World Health Organization, 2020). It also includes indoor recreational activities and relaxation exercises to daily practice (Kar et al., 2020; World Health Organization, 2020). To protect self and take care of self, it is also recommended that professionals ignore fake news and reduce social media (Kar et al., 2020; World Health Organization, 2020).

Self-care micro practices such as diaphragmatic respiration has shown an improvement in stress reduction; it is believed that the mechanism acts by increasing parasympathetic activation and, given that diaphragmatic breathing is low cost, self-administered, non-pharmacological, and highly portable, the practice is recommended (Fessell and Cherniss, 2020). To improve self-care, it is also necessary to act on self-efficacy, and the promotion of self-efficacy is recommended both for medical staff and for public; strengthening self-efficacy beliefs is recommended as strengthening beliefs about the disease include its severity and susceptibility (Mukhtar, 2020).

### Mental Health Services

Providing mental health services can be difficult during COVID-19, but such opportunities should be considered to prevent stress and burnout among professionals. Recommendations include building teams or multi-disciplinary teams of mental health experts who can provide mental health services or refer to appropriate resources if the health care worker shows signs of exhaustion (Sultana et al., 2020). Psychological counselors should be available in the staging areas of professionals to listen to staff difficulties and stories and provide support accordingly (Maben and Bridges, 2020; Shanafelt et al., 2020).

Group consultations or peer-support sessions are also recommended, which can allow specific topics to be dealt with in depth (Petzold et al., 2020; Sultana et al., 2020). Peer support and group support are particularly recommended among nurses for their “natural” tendency to take care of others and not themselves, which leads them to need others (colleagues and leaders) to remind them to think for themselves and to find ways to help new members feel safe, appreciated, and welcome as quickly as possible (Maben and Bridges, 2020).

### Digital Technologies

The use of digital interventions to improve health services and care outcomes is also recommended during COVID-19. The push toward digital is twofold. On the one hand, the use of electronic medical records and telemedicine can reduce the overloaded work experience (Moazzami et al., 2020; Sultana et al., 2020) among the frontline health care workers in COVID-19.

Another approach is to provide mental health resources and interventions that use digital platforms such as mobile phones, apps, or Internet devices. This can positively affect working and mental life and health professionals (Sultana et al., 2020). Due to increased assistance demands, the professional engagement of the operators should be guaranteed by implementing and providing psychological support services by phone of via the web (De Mei et al., 2020).

### Organizational Approaches

It is considered essential to improve organizational measures that affect the culture of work and stress in the workplace. Potential strategies include improving workflow management, organizing services focused on reducing workload, improving interoperability, organization of discussions and exchange of opinions, improvement of communication skills, providing adequate rest and exercise, and organizing seminars on coping skills (Sultana et al., 2020); such organizational support should include guarantees such as assistance to those doctors and nurses who fall ill, as well as medical and financial support for their families and protection from threats of neglect (Galbraith et al., 2020).

It is also recommended that the organization provide a resting place, guaranteed food and daily supplies, videos of their work to share with families to ease concerns, training to manage the patient’s psychological problems, and the provision of personal protective equipment (De Mei et al., 2020; Maben and Bridges, 2020; Shanafelt et al., 2020; World Health Organization, 2020). The recommendations regarding the implementation of all the necessary measures to protect the occupational safety suggest that the employer and the managers of the health structures must guarantee the adoption of preventive and protective measures, providing personal protective equipment in sufficient quantities for the health workers; this increases the sense of security and reduces stress (De Mei et al., 2020; Maben and Bridges, 2020; Petzold et al., 2020; World Health Organization, 2020).

The reports also suggest that regular and honest communication from the leaders of the organization toward the frontline professionals is essential, as well as the visibility and guarantee of access to physiological and safety needs (De Mei et al., 2020; Maben and Bridges, 2020; Petzold et al., 2020; World Health Organization, 2020). It is recommended that the organization create a series of communication channels (listening groups, e-mail suggestion box, town halls, and managers visiting hospital units) and ensure that the voice of health workers is part of the decision-making process (Shanafelt et al., 2020; World Health Organization, 2020).

The organization can also support health care professionals in addressing the mental health stigma in the workplace; creating a culture that encourages open communication and seeks to reduce the stigmatization of psychological vulnerability is recommended (Galbraith et al., 2020; Shanafelt et al., 2020).

### Evaluation of the Intervention

None of the 14 revised studies reported results of application of the intervention or recommendations.

Blake and others report the evaluation of the digital package tested. A total of 17,633 consultations of the tested package were carried out within 7 days of completion, and the evaluation (n. 55) indicated a high user satisfaction for content, usability, and utility (Blake et al., 2020).

Fessell and Cherniss (2020) recommended both physiologic bio-markers (blood pressure and salivary cortisol) and self-reported stress levels via the Depression Anxiety Stress Scales-21 as evaluation for diaphragmatic breathing.

## Discussion

Frontline health care providers during the COVID-19 pandemic were and are still being exposed to an enormous amount of stress for many reasons, such as lack of adequate protection, physical fatigue, exhausting shifts, and sometimes organizational difficulties, which can also include lack of human resources and being requested to operate out of one’s specialty out of necessity.

This rapid review was undertaken to assess the efficacy of stress reduction techniques for health care providers dealing with patients infected with severe coronavirus (SARS, MERS, and COVID-19). In particular, the authors were interested to identify what stress reduction techniques could be most effective in such particular circumstances.

Fourteen studies that fulfilled the selection criteria for this review were identified, 11 of which were recommendations. Only one intervention study met the quality appraisal criteria.

Only one intervention technique provided by a digital package using the Agile methodology (Blake et al., 2020) was described, and several recommended strategies based on individual, organizational, or team actions such as mindfulness, promotion of self-care, psychological counseling, digital platform information, and organizational support services were provided.

In the recommendations, it was specified that it is important to act on an organizational level. A lot of attention must be paid on communication, providing accurate updates in order to lower stress levels as much as possible, fostering a perception of control. All measures that guarantee the health care professionals’ safety and enhance their well-being must be put into place. In particular, attention to the organization of shifts, the creation of a safe place, and clear access to mental health services were outlined.

It was also specified that the interventions need to be tailor-made and safe; therefore, in the time of the peak of the pandemic, telehealth services must be provided. Some health care professionals seem to prefer not to have a direct contact with a mental health professional; therefore, resources, such as relaxation recordings and digital packaging, should be made available.

A number or self-care interventions were proposed, acting directly on essential needs, recreational activities, and specific stress reduction techniques, such as mindfulness-based interventions, diaphragmatic respiration, and acting on self-efficacy.

Even though these general indications were provided, no specific indications as to specific stress reduction techniques, which could be particularly effective during the COVID-19 pandemic, were provided. Further studies involving the health care providers themselves, and the measures of both satisfaction and effectiveness in reducing stress, with the use of patient-reported outcomes on anxiety, depression, PTSD, and insomnia are required.

### Limitations

Since a rapid review was conducted, there could bias as a consequence of streamlining the systematic review process. This bias could therefore have occurred during the selection of the studies, although three of the authors were involved in the selection in order to minimize this bias as much as possible.

## Conclusion

Frontline health care professionals are indispensable during pandemics such as COVID-19; therefore, there should be an important investment in order to safeguard their mental health and to lower their stress as much as possible. Further research is necessary to establish tailor-made effective stress reduction interventions for this population during these challenging and particular times.

## Author Contributions

EC proposed the study design and the idea, analyzed the literature and its development, and gave the final approval of the manuscript. BB and EB analyzed the literature and contributed to the preparation of the manuscript. VF and SP contributed to the shaping of the manuscript. ML revised and approved the final manuscript. All authors approved the submitted version.

## Conflict of Interest

The authors declare that the research was conducted in the absence of any commercial or financial relationships that could be construed as a potential conflict of interest.
