# Supplementary material for: Stress Reduction Techniques for Health Care Providers Dealing With Severe Coronavirus Infections (SARS, MERS, and COVID-19): A Rapid Review
Source: Front Psychol. 2020 Dec 10;11:589698. doi: 10.3389/fpsyg.2020.589698 (PMC7758192; doi:10.3389/fpsyg.2020.589698)
Supplement: Supplementary file 1 [file Data_Sheet_1.pdf]

SUPPLEMENTARY MATERIAL

Database Search Strings

PUBMED:

((((((((((((((((((((("Health Personnel"[MeSH Terms] OR ("health"[All Fields] AND "personnel"[All Fields])) OR "Health Personnel"[All Fields]) OR (("health"[All Fields] AND "care"[All Fields]) AND "personnel"[All Fields])) OR "health care personnel"[All Fields]) OR ((((("delivery of health care"[MeSH Terms] OR (("delivery"[All Fields] AND "health"[All Fields]) AND "care"[All Fields])) OR "delivery of health care"[All Fields]) OR ("health"[All Fields] AND "care"[All Fields])) OR "health care"[All Fields]) AND (("practitioner"[All Fields] OR "practitioner s"[All Fields]) OR "practitioners"[All Fields]))) OR (((("Health Personnel"[MeSH Terms] OR ("health"[All Fields] AND "personnel"[All Fields])) OR "Health Personnel"[All Fields]) OR (("health"[All Fields] AND "care"[All Fields]) AND "professional"[All Fields])) OR "health care professional"[All Fields])) OR (((("Health Personnel"[MeSH Terms] OR ("health"[All Fields] AND "personnel"[All Fields])) OR "Health Personnel"[All Fields]) OR (("health"[All Fields] AND "care"[All Fields]) AND "providers"[All Fields])) OR "health care providers"[All Fields])) OR (((("Health Personnel"[MeSH Terms] OR ("health"[All Fields] AND "personnel"[All Fields])) OR "Health Personnel"[All Fields]) OR (("health"[All Fields] AND "care"[All Fields]) AND "worker"[All Fields])) OR "health care worker"[All Fields])) OR (("Health Personnel"[MeSH Terms] OR ("health"[All Fields] AND "personnel"[All Fields])) OR "Health Personnel"[All Fields])) OR ((((("health occupations"[MeSH Terms] OR ("health"[All Fields] AND "occupations"[All Fields])) OR "health occupations"[All Fields]) OR ("health"[All Fields] AND "profession"[All Fields])) OR "health profession"[All Fields]) AND ((((("occupational groups"[MeSH Terms] OR ("occupational"[All Fields] AND "groups"[All Fields])) OR "occupational groups"[All Fields]) OR "personnel"[All Fields]) OR "personnel s"[All Fields]) OR "personnels"[All Fields]))) OR (((((("health"[MeSH Terms] OR "health"[All Fields]) OR "health s"[All Fields]) OR "healthful"[All Fields]) OR "healthfulness"[All Fields]) OR "healths"[All Fields]) AND ((((("occupational groups"[MeSH Terms] OR ("occupational"[All Fields] AND "groups"[All Fields])) OR "occupational groups"[All Fields]) OR "worker"[All Fields]) OR "workers"[All Fields]) OR "worker s"[All Fields]))) OR (((((("delivery of health care"[MeSH Terms] OR (("delivery"[All Fields] AND "health"[All Fields]) AND "care"[All Fields])) OR "delivery of health care"[All Fields]) OR "healthcare"[All Fields]) OR "healthcare s"[All Fields]) OR "healthcares"[All Fields]) AND ((((("occupational groups"[MeSH Terms] OR ("occupational"[All Fields] AND "groups"[All Fields])) OR "occupational groups"[All Fields]) OR "personnel"[All Fields]) OR "personnel s"[All Fields]) OR "personnels"[All Fields]))) OR (((((("delivery of health care"[MeSH Terms] OR (("delivery"[All Fields] AND "health"[All Fields]) AND "care"[All Fields])) OR "delivery of health care"[All Fields]) OR "healthcare"[All Fields]) OR "healthcare s"[All Fields]) OR "healthcares"[All Fields]) AND (("practitioner"[All Fields] OR "practitioner s"[All Fields]) OR "practitioners"[All Fields]))) OR (((("Health Personnel"[MeSH Terms] OR ("health"[All Fields] AND "personnel"[All Fields])) OR "Health Personnel"[All Fields]) OR ("healthcare"[All Fields] AND "professional"[All Fields])) OR "healthcare professional"[All Fields])) OR (((("Health Personnel"[MeSH Terms] OR ("health"[All Fields] AND "personnel"[All Fields])) OR "Health Personnel"[All Fields]) OR ("healthcare"[All Fields] AND "providers"[All Fields])) OR "healthcare providers"[All Fields])) OR (((("Health Personnel"[MeSH Terms] OR ("health"[All Fields] AND "personnel"[All Fields])) OR "Health Personnel"[All Fields]) OR ("healthcare"[All Fields] AND "worker"[All Fields])) OR "healthcare worker"[All Fields])) OR (("home health aides"[MeSH Terms] OR (("home"[All Fields] AND "health"[All Fields]) AND "aides"[All Fields])) OR "home health aides"[All Fields])) OR (((("Health Personnel"[MeSH Terms] OR ("health"[All Fields] AND "personnel"[All Fields])) OR "Health Personnel"[All Fields]) OR ("personnel"[All Fields] AND "health"[All Fields])) OR "personnel health"[All Fields])) OR

((("public health"[MeSH Terms] OR ("public"[All Fields] AND "health"[All Fields])) OR "public health"[All Fields]) AND ((((("office"[All Fields] OR "office s"[All Fields]) OR "officer"[All Fields]) OR "officer s"[All Fields]) OR "officers"[All Fields]) OR "offices"[All Fields]))) OR "Health Personnel"[MeSH Terms]) AND (((((("SARS"[All Fields] OR ((("coronavirus infections"[MeSH Terms] OR ("Coronavirus"[All Fields] AND "infections"[All Fields])) OR "coronavirus infections"[All Fields]) OR "mers"[All Fields])) OR (("Coronavirus"[MeSH Terms] OR "Coronavirus"[All Fields]) OR "coronaviruses"[All Fields])) OR ((((((("covid 19"[All Fields] OR "covid 2019"[All Fields]) OR "severe acute respiratory syndrome coronavirus 2"[Supplementary Concept]) OR "severe acute respiratory syndrome coronavirus 2"[All Fields]) OR "2019 ncov"[All Fields]) OR "sars cov 2"[All Fields]) OR "2019ncov"[All Fields]) OR (("wuhan"[All Fields] AND ("Coronavirus"[MeSH Terms] OR "Coronavirus"[All Fields])) AND (2019/12/1:2019/12/31[Date - Publication] OR 2020/1/1:2020/12/31[Date - Publication])))) OR (("severe acute respiratory syndrome"[MeSH Terms] OR ((("severe"[All Fields] AND "acute"[All Fields]) AND "respiratory"[All Fields]) AND "syndrome"[All Fields])) OR "severe acute respiratory syndrome"[All Fields])) OR (((("coronavirus infections"[MeSH Terms] OR ("Coronavirus"[All Fields] AND "infections"[All Fields])) OR "coronavirus infections"[All Fields]) OR ((("middle"[All Fields] AND "east"[All Fields]) AND "respiratory"[All Fields]) AND "syndrome"[All Fields])) OR "middle east respiratory syndrome"[All Fields])) OR (("Coronavirus"[MeSH Terms] OR "Middle East Respiratory Syndrome Coronavirus"[MeSH Terms]) OR "SARS Virus"[MeSH Terms]))) AND ((("stress"[All Fields] OR "stressed"[All Fields]) OR "stresses"[All Fields]) OR "stressful"[All Fields]) OR "stressfulness"[All Fields]) OR "stressing"[All Fields]) OR ((((("alarm"[All Fields] OR "alarm s"[All Fields]) OR "alarmed"[All Fields]) OR "alarming"[All Fields]) OR "alarms"[All Fields]) AND (("reaction"[All Fields] OR "reaction s"[All Fields]) OR "reactions"[All Fields]))) OR (((((("stress"[All Fields] OR "stressed"[All Fields]) OR "stresses"[All Fields]) OR "stressful"[All Fields]) OR "stressfulness"[All Fields]) OR "stressing"[All Fields]) AND ("capacities"[All Fields] OR "capacity"[All Fields]))) OR (((("fractures, stress"[MeSH Terms] OR ("fractures"[All Fields] AND "stress"[All Fields])) OR "stress fractures"[All Fields]) OR ("stress"[All Fields] AND "reaction"[All Fields])) OR "stress reaction"[All Fields])) OR (((((("stress"[All Fields] OR "stressed"[All Fields]) OR "stresses"[All Fields]) OR "stressful"[All Fields]) OR "stressfulness"[All Fields]) OR "stressing"[All Fields]) AND ((((((((((((((("resist"[All Fields] OR "resistance"[All Fields]) OR "resistances"[All Fields]) OR "resistant"[All Fields]) OR "resistants"[All Fields]) OR "resisted"[All Fields]) OR "resistence"[All Fields]) OR "resistences"[All Fields]) OR "resistent"[All Fields]) OR "resistibility"[All Fields]) OR "resisting"[All Fields]) OR "resistive"[All Fields]) OR "resistively"[All Fields]) OR "resistivities"[All Fields]) OR "resistivity"[All Fields]) OR "resists"[All Fields]))) OR (((((("stress"[All Fields] OR "stressed"[All Fields]) OR "stresses"[All Fields]) OR "stressful"[All Fields]) OR "stressfulness"[All Fields]) OR "stressing"[All Fields]) AND ((((((("response"[All Fields] OR "responses"[All Fields]) OR "responsive"[All Fields]) OR "responsiveness"[All Fields]) OR "responsivenesses"[All Fields]) OR "responsives"[All Fields]) OR "responsivities"[All Fields]) OR "responsivity"[All Fields]))) OR (((((("stress"[All Fields] OR "stressed"[All Fields]) OR "stresses"[All Fields]) OR "stressful"[All Fields]) OR "stressfulness"[All Fields]) OR "stressing"[All Fields]) AND ((((((("situate"[All Fields] OR "situated"[All Fields]) OR "situates"[All Fields]) OR "situating"[All Fields]) OR "situation"[All Fields]) OR "situation s"[All Fields]) OR "situational"[All Fields]) OR "situations"[All Fields]))) OR (((((("stress"[All Fields] OR "stressed"[All Fields]) OR "stresses"[All Fields]) OR "stressful"[All Fields]) OR "stressfulness"[All Fields]) OR "stressing"[All Fields]) AND (((((((((((((((((((((("immune tolerance"[MeSH Terms] OR ("immune"[All Fields] AND "tolerance"[All Fields])) OR "immune tolerance"[All Fields]) OR "tolerance"[All Fields]) OR "drug tolerance"[MeSH Terms]) OR ("drug"[All Fields] AND "tolerance"[All Fields])) OR "drug tolerance"[All Fields]) OR "tolerabilities"[All Fields]) OR "tolerability"[All Fields]) OR "tolerable"[All Fields]) OR "tolerableness"[All Fields]) OR "tolerably"[All Fields]) OR "tolerances"[All Fields]) OR "tolerant"[All Fields]) OR "tolerants"[All

Fields]) OR "tolerate"[All Fields]) OR "tolerated"[All Fields]) OR "tolerates"[All Fields]) OR "tolerating"[All Fields]) OR "toleration"[All Fields]) OR "tolerator"[All Fields]) OR "tolerators"[All Fields]) OR "tolerence"[All Fields]))) OR (((("Relaxation Therapy"[MeSH Terms] OR ("relaxation"[All Fields] AND "therapy"[All Fields])) OR "Relaxation Therapy"[All Fields]) OR ("relaxation"[All Fields] AND "training"[All Fields])) OR "relaxation training"[All Fields])) OR "mind-body"[All Fields]) AND (((("Relaxation Therapy"[MeSH Terms] OR ("relaxation"[All Fields] AND "therapy"[All Fields])) OR "Relaxation Therapy"[All Fields]) OR ("relaxation"[All Fields] AND "techniques"[All Fields])) OR "relaxation techniques"[All Fields])) OR ((((((((((("relaxant"[All Fields] OR "relaxants"[All Fields]) OR "relaxation"[MeSH Terms]) OR "relaxation"[All Fields]) OR "relax"[All Fields]) OR "relaxations"[All Fields]) OR "relaxed"[All Fields]) OR "relaxational"[All Fields]) OR "relaxative"[All Fields]) OR "relaxes"[All Fields]) OR "relaxing"[All Fields]) OR "relaxivities"[All Fields]) OR "relaxivity"[All Fields]) AND (((("method s"[All Fields] OR "methods"[MeSH Terms]) OR "methods"[All Fields]) OR "method"[All Fields]) OR "methods"[MeSH Subheading]))) OR (((("Relaxation Therapy"[MeSH Terms] OR ("relaxation"[All Fields] AND "therapy"[All Fields])) OR "Relaxation Therapy"[All Fields]) OR ("relaxation"[All Fields] AND "technics"[All Fields])) OR "relaxation technics"[All Fields])) OR (((("Relaxation Therapy"[MeSH Terms] OR ("relaxation"[All Fields] AND "therapy"[All Fields])) OR "Relaxation Therapy"[All Fields]) OR ("relaxation"[All Fields] AND "techniques"[All Fields])) OR "relaxation techniques"[All Fields])) OR (("Relaxation Therapy"[MeSH Terms] OR ("relaxation"[All Fields] AND "therapy"[All Fields])) OR "Relaxation Therapy"[All Fields])) OR (((("Relaxation Therapy"[MeSH Terms] OR ("relaxation"[All Fields] AND "therapy"[All Fields])) OR "Relaxation Therapy"[All Fields]) OR ("relaxation"[All Fields] AND "training"[All Fields])) OR "relaxation training"[All Fields])) OR ((((((((("education"[MeSH Subheading] OR "education"[All Fields]) OR "training"[All Fields]) OR "education"[MeSH Terms]) OR "train"[All Fields]) OR "train s"[All Fields]) OR "trained"[All Fields]) OR "training s"[All Fields]) OR "trainings"[All Fields]) OR "trains"[All Fields]) AND ((((((((((("relaxant"[All Fields] OR "relaxants"[All Fields]) OR "relaxation"[MeSH Terms]) OR "relaxation"[All Fields]) OR "relax"[All Fields]) OR "relaxations"[All Fields]) OR "relaxed"[All Fields]) OR "relaxational"[All Fields]) OR "relaxative"[All Fields]) OR "relaxes"[All Fields]) OR "relaxing"[All Fields]) OR "relaxivities"[All Fields]) OR "relaxivity"[All Fields]))) OR (((("breathing exercises"[MeSH Terms] OR ("breathing"[All Fields] AND "exercises"[All Fields])) OR "breathing exercises"[All Fields]) OR ("breathing"[All Fields] AND "exercise"[All Fields])) OR "breathing exercise"[All Fields])) OR (("breathing exercises"[MeSH Terms] OR ("breathing"[All Fields] AND "exercises"[All Fields])) OR "breathing exercises"[All Fields])) OR (((((((("breath"[All Fields] OR "breathe"[All Fields]) OR "breathed"[All Fields]) OR "breathes"[All Fields]) OR "breathings"[All Fields]) OR "breaths"[All Fields]) OR "respiration"[MeSH Terms]) OR "respiration"[All Fields]) OR "breathing"[All Fields]) AND (((((("therapeutics"[MeSH Terms] OR "therapeutics"[All Fields]) OR "therapies"[All Fields]) OR "therapy"[MeSH Subheading]) OR "therapy"[All Fields]) OR "therapy s"[All Fields]) OR "therapys"[All Fields]))) OR ((((("chested"[All Fields] OR "thorax"[MeSH Terms]) OR "thorax"[All Fields]) OR "chest"[All Fields]) OR "chests"[All Fields]) AND (((("physical therapy modalities"[MeSH Terms] OR (("physical"[All Fields] AND "therapy"[All Fields]) AND "modalities"[All Fields])) OR "physical therapy modalities"[All Fields]) OR ("physical"[All Fields] AND "therapy"[All Fields])) OR "physical therapy"[All Fields]))) OR ((((("chested"[All Fields] OR "thorax"[MeSH Terms]) OR "thorax"[All Fields]) OR "chest"[All Fields]) OR "chests"[All Fields]) AND (((("physical therapy modalities"[MeSH Terms] OR (("physical"[All Fields] AND "therapy"[All Fields]) AND "modalities"[All Fields])) OR "physical therapy modalities"[All Fields]) OR "physiotherapies"[All Fields]) OR "physiotherapy"[All Fields]))) OR (((("breathing exercises"[MeSH Terms] OR ("breathing"[All Fields] AND "exercises"[All Fields])) OR "breathing exercises"[All Fields]) OR ("exercise"[All Fields] AND "breathing"[All Fields])) OR "exercise breathing"[All Fields])) OR ((((((((((("exercise"[MeSH Terms] OR "exercise"[All Fields]) OR "exercises"[All Fields]) OR "exercise therapy"[MeSH Terms]) OR ("exercise"[All Fields] AND

"therapy"[All Fields])) OR "exercise therapy"[All Fields]) OR "exercise s"[All Fields]) OR "exercised"[All Fields]) OR "exerciser"[All Fields]) OR "exercisers"[All Fields]) OR "exercising"[All Fields]) AND "respiratory"[All Fields])) OR ((((((((((((("respiration"[MeSH Terms] OR "respiration"[All Fields]) OR "cell respiration"[MeSH Terms]) OR ("cell"[All Fields] AND "respiration"[All Fields])) OR "cell respiration"[All Fields]) OR "respirations"[All Fields]) OR "respirational"[All Fields]) OR "respirative"[All Fields]) OR "respiratively"[All Fields]) OR "respire"[All Fields]) OR "respired"[All Fields]) OR "respirer"[All Fields]) OR "respirers"[All Fields]) OR "respires"[All Fields]) OR "respiring"[All Fields]) AND (((((((((("exercise"[MeSH Terms] OR "exercise"[All Fields]) OR "exercises"[All Fields]) OR "exercise therapy"[MeSH Terms]) OR ("exercise"[All Fields] AND "therapy"[All Fields])) OR "exercise therapy"[All Fields]) OR "exercise s"[All Fields]) OR "exercised"[All Fields]) OR "exerciser"[All Fields]) OR "exercisers"[All Fields]) OR "exercising"[All Fields]))) OR ((((((((((((("respiration"[MeSH Terms] OR "respiration"[All Fields]) OR "cell respiration"[MeSH Terms]) OR ("cell"[All Fields] AND "respiration"[All Fields])) OR "cell respiration"[All Fields]) OR "respirations"[All Fields]) OR "respirational"[All Fields]) OR "respirative"[All Fields]) OR "respiratively"[All Fields]) OR "respire"[All Fields]) OR "respired"[All Fields]) OR "respirer"[All Fields]) OR "respirers"[All Fields]) OR "respires"[All Fields]) OR "respiring"[All Fields]) AND (((((("therapeutics"[MeSH Terms] OR "therapeutics"[All Fields]) OR "therapies"[All Fields]) OR "therapy"[MeSH Subheading]) OR "therapy"[All Fields]) OR "therapy s"[All Fields]) OR "therapys"[All Fields]))) OR ("respiratory"[All Fields] AND (((((((((("exercise"[MeSH Terms] OR "exercise"[All Fields]) OR "exercises"[All Fields]) OR "exercise therapy"[MeSH Terms]) OR ("exercise"[All Fields] AND "therapy"[All Fields])) OR "exercise therapy"[All Fields]) OR "exercise s"[All Fields]) OR "exercised"[All Fields]) OR "exerciser"[All Fields]) OR "exercisers"[All Fields]) OR "exercising"[All Fields]))) OR ("respiratory"[All Fields] AND (((("physical therapy modalities"[MeSH Terms] OR (("physical"[All Fields] AND "therapy"[All Fields]) AND "modalities"[All Fields])) OR "physical therapy modalities"[All Fields]) OR "physiotherapies"[All Fields]) OR "physiotherapy"[All Fields]))) OR ((((("psychologie"[All Fields] OR "psychologies"[All Fields]) OR "psychology"[MeSH Subheading]) OR "psychology"[All Fields]) OR "psychology"[MeSH Terms]) OR "psychology s"[All Fields])) OR (("cognitive science"[MeSH Terms] OR ("cognitive"[All Fields] AND "science"[All Fields])) OR "cognitive science"[All Fields])) OR ((((("psychologie"[All Fields] OR "psychologies"[All Fields]) OR "psychology"[MeSH Subheading]) OR "psychology"[All Fields]) OR "psychology"[MeSH Terms]) OR "psychology s"[All Fields])) OR (((("psychology, applied"[MeSH Terms] OR ("psychology"[All Fields] AND "applied"[All Fields])) OR "applied psychology"[All Fields]) OR ("psychology"[All Fields] AND "applied"[All Fields])) OR "psychology applied"[All Fields])) OR (((("psychology, comparative"[MeSH Terms] OR ("psychology"[All Fields] AND "comparative"[All Fields])) OR "comparative psychology"[All Fields]) OR ("psychology"[All Fields] AND "comparative"[All Fields])) OR "psychology comparative"[All Fields])) OR (((("psychology, educational"[MeSH Terms] OR ("psychology"[All Fields] AND "educational"[All Fields])) OR "educational psychology"[All Fields]) OR ("psychology"[All Fields] AND "educational"[All Fields])) OR "psychology educational"[All Fields])) OR (((("psychology, experimental"[MeSH Terms] OR ("psychology"[All Fields] AND "experimental"[All Fields])) OR "experimental psychology"[All Fields]) OR ("psychology"[All Fields] AND "experimental"[All Fields])) OR "psychology experimental"[All Fields])) OR (((("psychology, industrial"[MeSH Terms] OR ("psychology"[All Fields] AND "industrial"[All Fields])) OR "industrial psychology"[All Fields]) OR ("psychology"[All Fields] AND "industrial"[All Fields])) OR "psychology industrial"[All Fields])) OR (((("psychology, military"[MeSH Terms] OR ("psychology"[All Fields] AND "military"[All Fields])) OR "military psychology"[All Fields]) OR ("psychology"[All Fields] AND "military"[All Fields])) OR "psychology military"[All Fields])) OR (("schizophrenic psychology"[MeSH Terms] OR ("schizophrenic"[All Fields] AND "psychology"[All Fields])) OR "schizophrenic psychology"[All Fields])) OR (((((("mind s"[All Fields] OR "minded"[All Fields]) OR "mindful"[All

Fields]) OR "mindfulness"[MeSH Terms]) OR "mindfulness"[All Fields]) OR "minding"[All Fields]) OR "minds"[All Fields])) OR (((((("mind s"[All Fields] OR "minded"[All Fields]) OR "mindful"[All Fields]) OR "mindfulness"[MeSH Terms]) OR "mindfulness"[All Fields]) OR "minding"[All Fields]) OR "minds"[All Fields])) OR (((("complementary therapies"[MeSH Terms] OR ("complementary"[All Fields] AND "therapies"[All Fields])) OR "complementary therapies"[All Fields]) OR ("alternative"[All Fields] AND "medicine"[All Fields])) OR "alternative medicine"[All Fields])) OR (((("complementary therapies"[MeSH Terms] OR ("complementary"[All Fields] AND "therapies"[All Fields])) OR "complementary therapies"[All Fields]) OR ("alternative"[All Fields] AND "medicine"[All Fields])) OR "alternative medicine"[All Fields])) OR (((("complementary therapies"[MeSH Terms] OR ("complementary"[All Fields] AND "therapies"[All Fields])) OR "complementary therapies"[All Fields]) OR ("alternative"[All Fields] AND "therapies"[All Fields])) OR "alternative therapies"[All Fields])) OR (((("complementary therapies"[MeSH Terms] OR ("complementary"[All Fields] AND "therapies"[All Fields])) OR "complementary therapies"[All Fields]) OR ("alternative"[All Fields] AND "therapy"[All Fields])) OR "alternative therapy"[All Fields])) OR (((("complementary therapies"[MeSH Terms] OR ("complementary"[All Fields] AND "therapies"[All Fields])) OR "complementary therapies"[All Fields]) OR ("complementary"[All Fields] AND "medicine"[All Fields])) OR "complementary medicine"[All Fields])) OR (("complementary therapies"[MeSH Terms] OR ("complementary"[All Fields] AND "therapies"[All Fields])) OR "complementary therapies"[All Fields])) OR (("diet fads"[MeSH Terms] OR ("diet"[All Fields] AND "fads"[All Fields])) OR "diet fads"[All Fields])) OR (((("eclecticism, historical"[MeSH Terms] OR ("eclecticism"[All Fields] AND "historical"[All Fields])) OR "historical eclecticism"[All Fields]) OR ("eclecticism"[All Fields] AND "historical"[All Fields])) OR "eclecticism historical"[All Fields])) OR (("mental healing"[MeSH Terms] OR ("mental"[All Fields] AND "healing"[All Fields])) OR "mental healing"[All Fields])) OR ((("sci am mind"[Journal] OR "mind"[Journal]) OR "mind"[All Fields]) AND ((("human body"[MeSH Terms] OR ("human"[All Fields] AND "body"[All Fields])) OR "human body"[All Fields]) OR "body"[All Fields]) AND ((((("methods"[MeSH Subheading] OR "methods"[All Fields]) OR "techniques"[All Fields]) OR "methods"[MeSH Terms]) OR "technique"[All Fields]) OR "technique s"[All Fields]))) OR (((("mind-body therapies"[MeSH Terms] OR ("mind-body"[All Fields] AND "therapies"[All Fields])) OR "mind body therapies"[All Fields]) OR (("mind"[All Fields] AND "body"[All Fields]) AND "therapies"[All Fields])) OR "mind body therapies"[All Fields])) OR (((("mind-body therapies"[MeSH Terms] OR ("mind-body"[All Fields] AND "therapies"[All Fields])) OR "mind body therapies"[All Fields]) OR (("mind"[All Fields] AND "body"[All Fields]) AND "therapy"[All Fields])) OR "mind body therapy"[All Fields])) OR ("mind-body"[All Fields] AND ((((((((("family"[MeSH Terms] OR "family"[All Fields]) OR "relation"[All Fields]) OR "relatability"[All Fields]) OR "relatable"[All Fields]) OR "related"[All Fields]) OR "relates"[All Fields]) OR "relating"[All Fields]) OR "relational"[All Fields]) OR "relations"[All Fields]) AND ((((((("metaphyseal"[All Fields] OR "metaphyseally"[All Fields]) OR "metaphyses"[All Fields]) OR "metaphysical"[All Fields]) OR "metaphysically"[All Fields]) OR "metaphysics"[MeSH Terms]) OR "metaphysics"[All Fields]) OR "metaphysic"[All Fields]))) OR (((("mind-body therapies"[MeSH Terms] OR ("mind-body"[All Fields] AND "therapies"[All Fields])) OR "mind body therapies"[All Fields]) OR (("mind"[All Fields] AND "body"[All Fields]) AND "therapies"[All Fields])) OR "mind body therapies"[All Fields])) OR (((((((((((((((((("polar"[All Fields] OR "polarisabilities"[All Fields]) OR "polarisability"[All Fields]) OR "polarisable"[All Fields]) OR "polarisation"[All Fields]) OR "polarisations"[All Fields]) OR "polarise"[All Fields]) OR "polarised"[All Fields]) OR "polarising"[All Fields]) OR "polarities"[All Fields]) OR "polarity"[All Fields]) OR "polarization"[All Fields]) OR "polarizations"[All Fields]) OR "polarize"[All Fields]) OR "polarized"[All Fields]) OR "polarizer"[All Fields]) OR "polarizers"[All Fields]) OR "polarizes"[All Fields]) OR "polarizing"[All Fields]) OR "polars"[All Fields]) AND (((((("therapeutics"[MeSH Terms] OR "therapeutics"[All Fields]) OR "therapies"[All Fields]) OR "therapy"[MeSH Subheading]) OR "therapy"[All Fields]) OR "therapy s"[All Fields]) OR "therapys"[All Fields])))

OR ("radiesthesia"[MeSH Terms] OR "radiesthesia"[All Fields])) OR ("reflexotherapy"[MeSH Terms] OR "reflexotherapy"[All Fields])) OR (((((("therapeutical"[All Fields] OR "therapeutically"[All Fields]) OR "therapeuticals"[All Fields]) OR "therapeutics"[MeSH Terms]) OR "therapeutics"[All Fields]) OR "therapeutic"[All Fields]) AND "cults"[All Fields])) OR (((((((((("meditate"[All Fields] OR "meditated"[All Fields]) OR "meditating"[All Fields]) OR "meditation"[MeSH Terms]) OR "meditation"[All Fields]) OR "meditations"[All Fields])) OR "meditational"[All Fields]) OR "meditative"[All Fields]) OR "meditator"[All Fields]) OR "meditators"[All Fields])) OR (((("psychotherapie"[All Fields] OR "psychotherapy"[MeSH Terms]) OR "psychotherapy"[All Fields]) OR "psychotherapies"[All Fields]) OR "psychotherapy s"[All Fields])) OR (("holistic"[All Fields] OR "holistically"[All Fields]) AND (((("psychotherapie"[All Fields] OR "psychotherapy"[MeSH Terms]) OR "psychotherapy"[All Fields]) OR "psychotherapies"[All Fields]) OR "psychotherapy s"[All Fields]))) OR (("psychotherapeutic processes"[MeSH Terms] OR ("psychotherapeutic"[All Fields] AND "processes"[All Fields])) OR "psychotherapeutic processes"[All Fields])) OR (((((((("psychotherapeutical"[All Fields] OR "psychotherapeutically"[All Fields]) OR "psychotropic drugs"[Pharmacological Action]) OR "psychotropic drugs"[MeSH Terms]) OR ("psychotropic"[All Fields] AND "drugs"[All Fields])) OR "psychotropic drugs"[All Fields]) OR "psychotherapeutic"[All Fields]) OR "psychotherapeutics"[All Fields]) AND ((((((((("education"[MeSH Subheading] OR "education"[All Fields]) OR "training"[All Fields]) OR "education"[MeSH Terms]) OR "train"[All Fields]) OR "train s"[All Fields]) OR "trained"[All Fields]) OR "training s"[All Fields]) OR "trainings"[All Fields]) OR "trains"[All Fields]))) OR (((("psychotherapie"[All Fields] OR "psychotherapy"[MeSH Terms]) OR "psychotherapy"[All Fields]) OR "psychotherapies"[All Fields]) OR "psychotherapy s"[All Fields])) OR (((("psychotherapy, multiple"[MeSH Terms] OR ("psychotherapy"[All Fields] AND "multiple"[All Fields])) OR "multiple psychotherapy"[All Fields]) OR ("psychotherapy"[All Fields] AND "multiple"[All Fields])) OR "psychotherapy multiple"[All Fields])) OR (("socioenvironmental therapy"[MeSH Terms] OR ("socioenvironmental"[All Fields] AND "therapy"[All Fields])) OR "socioenvironmental therapy"[All Fields])) OR (("mental health"[MeSH Terms] OR ("mental"[All Fields] AND "health"[All Fields])) OR "mental health"[All Fields])) OR ((((("condition s"[All Fields] OR "conditions"[All Fields]) OR "disease"[MeSH Terms]) OR "disease"[All Fields]) OR "condition"[All Fields]) AND (((((((("mental"[All Fields] OR "mentalities"[All Fields]) OR "mentality"[All Fields]) OR "mentalization"[MeSH Terms]) OR "mentalization"[All Fields]) OR "mentalizing"[All Fields]) OR "mentalize"[All Fields]) OR "mentalized"[All Fields]) OR "mentally"[All Fields]))) OR (((("mental health"[MeSH Terms] OR ("mental"[All Fields] AND "health"[All Fields])) OR "mental health"[All Fields]) OR ("health"[All Fields] AND "mental"[All Fields])) OR "health mental"[All Fields])) OR ((((((((("mental"[All Fields] OR "mentalities"[All Fields]) OR "mentality"[All Fields]) OR "mentalization"[MeSH Terms]) OR "mentalization"[All Fields]) OR "mentalizing"[All Fields]) OR "mentalize"[All Fields]) OR "mentalized"[All Fields]) OR "mentally"[All Fields]) AND "care"[All Fields])) OR ((((((((("mental"[All Fields] OR "mentalities"[All Fields]) OR "mentality"[All Fields]) OR "mentalization"[MeSH Terms]) OR "mentalization"[All Fields]) OR "mentalizing"[All Fields]) OR "mentalize"[All Fields]) OR "mentalized"[All Fields]) OR "mentally"[All Fields]) AND (((("condition s"[All Fields] OR "conditions"[All Fields]) OR "disease"[MeSH Terms]) OR "disease"[All Fields]) OR "condition"[All Fields]))) OR ((((((((("mental"[All Fields] OR "mentalities"[All Fields]) OR "mentality"[All Fields]) OR "mentalization"[MeSH Terms]) OR "mentalization"[All Fields]) OR "mentalizing"[All Fields]) OR "mentalize"[All Fields]) OR "mentalized"[All Fields]) OR "mentally"[All Fields]) AND (("factor"[All Fields] OR "factor s"[All Fields]) OR "factors"[All Fields]))) OR (("mental health"[MeSH Terms] OR ("mental"[All Fields] AND "health"[All Fields])) OR "mental health"[All Fields])) OR ((((((((("mental"[All Fields] OR "mentalities"[All Fields]) OR "mentality"[All Fields]) OR "mentalization"[MeSH Terms]) OR "mentalization"[All Fields]) OR "mentalizing"[All Fields]) OR "mentalize"[All Fields]) OR "mentalized"[All Fields]) OR "mentally"[All Fields]) AND "help"[All Fields])) OR ((((((((("mental"[All Fields] OR "mentalities"[All Fields]) OR

"mentality"[All Fields]) OR "mentalization"[MeSH Terms]) OR "mentalization"[All Fields]) OR "mentalizing"[All Fields]) OR "mentalize"[All Fields]) OR "mentalized"[All Fields]) OR "mentally"[All Fields]) AND ((((("service"[All Fields] OR "service s"[All Fields]) OR "serviced"[All Fields]) OR "services"[All Fields]) OR "services s"[All Fields]) OR "servicing"[All Fields]))) OR (((((((("mental"[All Fields] OR "mentalities"[All Fields]) OR "mentality"[All Fields]) OR "mentalization"[MeSH Terms]) OR "mentalization"[All Fields]) OR "mentalizing"[All Fields]) OR "mentalize"[All Fields]) OR "mentalized"[All Fields]) OR "mentally"[All Fields]) AND (((("state"[All Fields] OR "state s"[All Fields]) OR "stated"[All Fields]) OR "states"[All Fields]) OR "stating"[All Fields]))) OR (((((((("mental"[All Fields] OR "mentalities"[All Fields]) OR "mentality"[All Fields]) OR "mentalization"[MeSH Terms]) OR "mentalization"[All Fields]) OR "mentalizing"[All Fields]) OR "mentalize"[All Fields]) OR "mentalized"[All Fields]) OR "mentally"[All Fields]) AND "status"[All Fields])) OR (("mental status schedule"[MeSH Terms] OR (("mental"[All Fields] AND "status"[All Fields]) AND "schedule"[All Fields])) OR "mental status schedule"[All Fields])) OR (((("psychic"[All Fields] OR "psychical"[All Fields]) OR "psychically"[All Fields]) OR "psychics"[All Fields]) AND ((((("health"[MeSH Terms] OR "health"[All Fields]) OR "health s"[All Fields]) OR "healthful"[All Fields]) OR "healthfulness"[All Fields]) OR "healths"[All Fields]))) OR ((("anxiety"[MeSH Terms] OR "anxiety"[All Fields]) OR "anxieties"[All Fields]) OR "anxiety s"[All Fields])) OR ((((((((((((((("consciousness disorders"[MeSH Terms] OR ("consciousness"[All Fields] AND "disorders"[All Fields])) OR "consciousness disorders"[All Fields]) OR "depressed"[All Fields]) OR "depression"[MeSH Terms]) OR "depression"[All Fields]) OR "depressions"[All Fields]) OR "depression s"[All Fields]) OR "depressive disorder"[MeSH Terms]) OR ("depressive"[All Fields] AND "disorder"[All Fields])) OR "depressive disorder"[All Fields]) OR "depressivity"[All Fields]) OR "depressive"[All Fields]) OR "depressively"[All Fields]) OR "depressiveness"[All Fields]) OR "depressives"[All Fields]) AND ((((("psychologie"[All Fields] OR "psychologies"[All Fields]) OR "psychology"[MeSH Subheading]) OR "psychology"[All Fields]) OR "psychology"[MeSH Terms]) OR "psychology s"[All Fields]))) ((((("sleep initiation and maintenance disorders"[MeSH Terms]) OR ((("sleep"[All Fields] AND "initiation"[All Fields]) AND "maintenance"[All Fields]) AND "disorders"[All Fields])) OR "sleep initiation and maintenance disorders"[All Fields]) OR "insomnia"[All Fields]) OR "insomnias"[All Fields])) OR "agrypnia"[All Fields]) OR "hyposomnia"[All Fields]) ((((("sleep initiation and maintenance disorders"[MeSH Terms]) OR ((("sleep"[All Fields] AND "initiation"[All Fields]) AND "maintenance"[All Fields]) AND "disorders"[All Fields])) OR "sleep initiation and maintenance disorders"[All Fields]) OR "insomnia"[All Fields]) OR "insomnias"[All Fields])) OR ((((("sleep"[MeSH Terms] OR "sleep"[All Fields]) OR "sleeping"[All Fields]) OR "sleeps"[All Fields]) OR "sleep s"[All Fields]) AND (((((((((("initial"[All Fields] OR "initially"[All Fields]) OR "initials"[All Fields]) OR "initiate"[All Fields]) OR "initiated"[All Fields]) OR "initiates"[All Fields]) OR "initiating"[All Fields]) OR "initiation"[All Fields]) OR "initiations"[All Fields]) OR "initiator"[All Fields]) OR "initiators"[All Fields]))) AND ((("maintenance"[MeSH Terms] OR "maintenance"[All Fields]) OR "maintenances"[All Fields]) AND ((((("disease"[MeSH Terms] OR "disease"[All Fields]) OR "disorder"[All Fields]) OR "disorders"[All Fields]) OR "disorder s"[All Fields]) OR "disordes"[All Fields]))) OR (((("sleep initiation and maintenance disorders"[MeSH Terms] OR ((("sleep"[All Fields] AND "initiation"[All Fields]) AND "maintenance"[All Fields]) AND "disorders"[All Fields])) OR "sleep initiation and maintenance disorders"[All Fields]) OR "sleeplessness"[All Fields]) OR "sleepless"[All Fields])) OR (((("stress disorders, post-traumatic"[MeSH Terms] OR (("stress"[All Fields] AND "disorders"[All Fields]) AND "post-traumatic"[All Fields])) OR "post-traumatic stress disorders"[All Fields]) OR (("posttraumatic"[All Fields] AND "stress"[All Fields]) AND "disorder"[All Fields])) OR "posttraumatic stress disorder"[All Fields])) OR ((("stress disorders, post-traumatic"[MeSH Terms] OR (("stress"[All Fields] AND "disorders"[All Fields]) AND "post-traumatic"[All Fields])) OR "post-traumatic stress disorders"[All Fields]) OR "ptsd"[All Fields])) OR (("combat disorders"[MeSH Terms] OR ("combat"[All Fields] AND "disorders"[All Fields])) OR "combat disorders"[All Fields])) OR

(((("stress disorders, post-traumatic"[MeSH Terms] OR (("stress"[All Fields] AND "disorders"[All Fields]) AND "post-traumatic"[All Fields])) OR "post-traumatic stress disorders"[All Fields]) OR ("combat"[All Fields] AND "fatigue"[All Fields])) OR "combat fatigue"[All Fields])) OR (((((((("combat"[All Fields] OR "combatant"[All Fields]) OR "combatants"[All Fields]) OR "combated"[All Fields]) OR "combating"[All Fields]) OR "combats"[All Fields]) OR "combatted"[All Fields]) OR "combatting"[All Fields]) AND ((((("stress"[All Fields] OR "stressed"[All Fields]) OR "stresses"[All Fields]) OR "stressful"[All Fields]) OR "stressfulness"[All Fields]) OR "stressing"[All Fields]))) OR ("post-traumatic"[All Fields] AND ((((("stress"[All Fields] OR "stressed"[All Fields]) OR "stresses"[All Fields]) OR "stressful"[All Fields]) OR "stressfulness"[All Fields]) OR "stressing"[All Fields]))) OR (((("stress disorders, post-traumatic"[MeSH Terms] OR (("stress"[All Fields] AND "disorders"[All Fields]) AND "post-traumatic"[All Fields])) OR "post-traumatic stress disorders"[All Fields]) OR ((("post"[All Fields] AND "traumatic"[All Fields]) AND "stress"[All Fields]) AND "disorder"[All Fields])) OR "post traumatic stress disorder"[All Fields])) OR ("posttraumatic"[All Fields] AND ((("neurotic disorders"[MeSH Terms] OR ("neurotic"[All Fields] AND "disorders"[All Fields])) OR "neurotic disorders"[All Fields]) OR "neurosis"[All Fields]))) OR ("posttraumatic"[All Fields] AND ((("psychic"[All Fields] OR "psychical"[All Fields]) OR "psychically"[All Fields]) OR "psychics"[All Fields]) AND (((((((("syndrom"[All Fields] OR "syndromal"[All Fields]) OR "syndromally"[All Fields]) OR "syndrome"[MeSH Terms]) OR "syndrome"[All Fields]) OR "syndromes"[All Fields]) OR "syndrome s"[All Fields]) OR "syndromic"[All Fields]) OR "syndroms"[All Fields]))) OR ("posttraumatic"[All Fields] AND ((("psychotic disorders"[MeSH Terms] OR ("psychotic"[All Fields] AND "disorders"[All Fields])) OR "psychotic disorders"[All Fields]) OR "psychosis"[All Fields]))) OR ("posttraumatic"[All Fields] AND ((((("stress"[All Fields] OR "stressed"[All Fields]) OR "stresses"[All Fields]) OR "stressful"[All Fields]) OR "stressfulness"[All Fields]) OR "stressing"[All Fields]))) OR (((("stress disorders, post-traumatic"[MeSH Terms] OR (("stress"[All Fields] AND "disorders"[All Fields]) AND "post-traumatic"[All Fields])) OR "post-traumatic stress disorders"[All Fields]) OR (("posttraumatic"[All Fields] AND "stress"[All Fields]) AND "disorder"[All Fields])) OR "posttraumatic stress disorder"[All Fields])) OR ("posttraumatic"[All Fields] AND (((((((("syndrom"[All Fields] OR "syndromal"[All Fields]) OR "syndromally"[All Fields]) OR "syndrome"[MeSH Terms]) OR "syndrome"[All Fields]) OR "syndromes"[All Fields]) OR "syndrome s"[All Fields]) OR "syndromic"[All Fields]) OR "syndroms"[All Fields]))) OR (((("psychotic disorders"[MeSH Terms] OR ("psychotic"[All Fields] AND "disorders"[All Fields])) OR "psychotic disorders"[All Fields]) OR "psychosis"[All Fields]) AND "posttraumatic"[All Fields])) OR ((((("stress disorders, traumatic"[MeSH Terms] OR (("stress"[All Fields] AND "disorders"[All Fields]) AND "traumatic"[All Fields])) OR "traumatic stress disorders"[All Fields]) OR ("stress"[All Fields] AND "disorders"[All Fields])) OR "stress disorders"[All Fields]) AND "post-traumatic"[All Fields])) OR (((("stress disorders, traumatic"[MeSH Terms] OR (("stress"[All Fields] AND "disorders"[All Fields]) AND "traumatic"[All Fields])) OR "traumatic stress disorders"[All Fields]) OR (("stress"[All Fields] AND "disorders"[All Fields]) AND "traumatic"[All Fields])) OR "stress disorders traumatic"[All Fields])) OR (((((("stress"[All Fields] OR "stressed"[All Fields]) OR "stresses"[All Fields]) OR "stressful"[All Fields]) OR "stressfulness"[All Fields]) OR "stressing"[All Fields]) AND "posttraumatic"[All Fields])) OR ((((((("injuries"[MeSH Subheading] OR "injuries"[All Fields]) OR "trauma"[All Fields]) OR "wounds and injuries"[MeSH Terms]) OR ("wounds"[All Fields] AND "injuries"[All Fields])) OR "wounds and injuries"[All Fields]) OR "trauma s"[All Fields]) OR "traumas"[All Fields])) AND ((("stressor"[All Fields] OR "stressor s"[All Fields]) OR "stressors"[All Fields]) AND (((((((("family"[MeSH Terms] OR "family"[All Fields]) OR "relation"[All Fields]) OR "relatability"[All Fields]) OR "relatable"[All Fields]) OR "related"[All Fields]) OR "relates"[All Fields]) OR "relating"[All Fields]) OR "relational"[All Fields]) OR "relations"[All Fields]) AND ((((("disease"[MeSH Terms] OR "disease"[All Fields]) OR "disorder"[All Fields]) OR "disorders"[All Fields]) OR "disorder s"[All Fields]) OR "disordes"[All Fields]))) OR

((((((((("traumatic"[All Fields] OR "traumatically"[All Fields]) OR "traumatism"[All Fields]) OR "traumatisms"[All Fields]) OR "traumatization"[All Fields]) OR "traumatizations"[All Fields]) OR "traumatize"[All Fields]) OR "traumatized"[All Fields]) OR "traumatizes"[All Fields]) OR "traumatizing"[All Fields]) AND ((((("stress"[All Fields] OR "stressed"[All Fields]) OR "stresses"[All Fields]) OR "stressful"[All Fields]) OR "stressfulness"[All Fields]) OR "stressing"[All Fields]))) OR (((("stress disorders, traumatic"[MeSH Terms] OR (("stress"[All Fields] AND "disorders"[All Fields]) AND "traumatic"[All Fields])) OR "traumatic stress disorders"[All Fields]) OR (("traumatic"[All Fields] AND "stress"[All Fields]) AND "disorder"[All Fields])) OR "traumatic stress disorder"[All Fields])) OR (((("combat disorders"[MeSH Terms] OR ("combat"[All Fields] AND "disorders"[All Fields])) OR "combat disorders"[All Fields]) OR ("war"[All Fields] AND "neurosis"[All Fields])) OR "war neurosis"[All Fields])) OR ((((("burnout s"[All Fields] OR "burnout, psychological"[MeSH Terms]) OR ("burnout"[All Fields] AND "psychological"[All Fields])) OR "psychological burnout"[All Fields]) OR "burnout"[All Fields]) OR "burnouts"[All Fields])) OR (((("burnout, psychological"[MeSH Terms] OR ("burnout"[All Fields] AND "psychological"[All Fields])) OR "psychological burnout"[All Fields]) OR ("burn"[All Fields] AND "out"[All Fields])) OR "burn out"[All Fields])) OR (((("burnout, psychological"[MeSH Terms] OR ("burnout"[All Fields] AND "psychological"[All Fields])) OR "psychological burnout"[All Fields]) OR (("burn"[All Fields] AND "out"[All Fields]) AND "syndrome"[All Fields])) OR "burn out syndrome"[All Fields])) OR (((("burnout, psychological"[MeSH Terms] OR ("burnout"[All Fields] AND "psychological"[All Fields])) OR "psychological burnout"[All Fields]) OR ("burnout"[All Fields] AND "syndrome"[All Fields])) OR "burnout syndrome"[All Fields])) OR (((("burnout, psychological"[MeSH Terms] OR ("burnout"[All Fields] AND "psychological"[All Fields])) OR "psychological burnout"[All Fields]) OR ("burnout"[All Fields] AND "psychological"[All Fields])) OR "burnout psychological"[All Fields])) OR (((("burnout, psychological"[MeSH Terms] OR ("burnout"[All Fields] AND "psychological"[All Fields])) OR "psychological burnout"[All Fields]) OR (("psychological"[All Fields] AND "burn"[All Fields]) AND "out"[All Fields])) OR "psychological burn out"[All Fields])) OR ((("burnout, psychological"[MeSH Terms] OR ("burnout"[All Fields] AND "psychological"[All Fields])) OR "psychological burnout"[All Fields]) OR ("psychological"[All Fields] AND "burnout"[All Fields]))) OR ((((("social stigma"[MeSH Terms] OR ("social"[All Fields] AND "stigma"[All Fields])) OR "social stigma"[All Fields]) OR "stigma"[All Fields]) OR "stigmas"[All Fields]) OR "stigma s"[All Fields])) OR (("quality of life"[MeSH Terms] OR ("quality"[All Fields] AND "life"[All Fields])) OR "quality of life"[All Fields])) OR "hrql"[All Fields]) OR (((("quality of life"[MeSH Terms] OR ("quality"[All Fields] AND "life"[All Fields])) OR "quality of life"[All Fields]) OR ((("health"[All Fields] AND "related"[All Fields]) AND "quality"[All Fields]) AND "life"[All Fields])) OR "health related quality of life"[All Fields])) OR (((("quality of life"[MeSH Terms] OR ("quality"[All Fields] AND "life"[All Fields])) OR "quality of life"[All Fields]) OR ("life"[All Fields] AND "quality"[All Fields])) OR "life quality"[All Fields])) OR (("quality of life"[MeSH Terms] OR ("quality"[All Fields] AND "life"[All Fields])) OR "quality of life"[All Fields])) OR ((("stress disorders, traumatic, acute"[MeSH Terms] OR "stress disorders, post-traumatic"[MeSH Terms]) OR "stress disorders, traumatic"[MeSH Terms]) OR "stress, physiological"[MeSH Terms])) OR "Relaxation Therapy"[MeSH Terms]) OR "Psychological Techniques"[MeSH Terms])

PSYCHINFO:

(((((((stress or alarm reaction or stress capacity or stress reaction or stress resistance or stress response or stress situation or stress tolerance or relaxation training or mind-body) and relaxation techniques)

or relaxation method or relaxation technics or relaxation techniques or relaxation therapy or relaxation training or training, relaxation or breathing exercise or breathing exercises or breathing therapy or chest physical therapy or chest physiotherapy or exercise, breathing or exercise, respiratory or respiration exercise or respiration therapy or respiratory exercise or respiratory physiotherapy or psychology or cognitive science or psychology or psychology, applied or psychology, comparative or psychology, educational or psychology, experimental or psychology, industrial or psychology, military or schizophrenic psychology or mindfulness or mindfulness or alternative medicine or alternative medicine or alternative therapies or alternative therapy or complementary medicine or complementary therapies or diet fads or eclecticism, historical or mental healing or mind body technique or mind body therapies or mind body therapy or mind-body relations, metaphysical or mind-body therapies or polarity therapy or radiaesthesia or radiesthesia or reflexotherapy or therapeutic cults or meditation or psychotherapy or holistic psychotherapy or psychotherapeutic processes or psychotherapeutic training or psychotherapy or psychotherapy, multiple or socioenvironmental therapy or mental health or condition, mental or health, mental or mental care or mental condition or mental factor or mental health or mental help or mental service or mental state or mental status or mental status schedule or psychic health or anxiety or depression psychology or insomnia or agrypnia or hyposomnia or insomnia or sleep initiation) and maintenance disorders) or sleeplessness or posttraumatic stress disorder or ptsd or combat disorders or combat fatigue or combat stress or post-traumatic stress or post-traumatic stress disorder or posttraumatic neurosis or posttraumatic psychic syndrome or posttraumatic psychosis or posttraumatic stress or posttraumatic stress disorder or posttraumatic syndrome or psychosis, posttraumatic or stress disorders, post-traumatic or stress disorders, traumatic or stress, posttraumatic or trauma) and stressor related disorders) or traumatic stress or traumatic stress disorder or war neurosis or burnout or burn-out or burn-out syndrome or burnout syndrome or burnout, psychological or psychological burn-out or psychological burnout or stigma or quality of life or hrql or health related quality of life or life quality or quality of life) AND (SARS or MERS or CORONAVIRUS or COVID 19 or severe acute respiratory syndrome OR middle east respiratory syndrome) AND (health care personnel or health care practitioner or health care professional or health care provider or health care worker or health personnel or health profession personnel or health worker or healthcare personnel or healthcare practitioner or healthcare professional or healthcare provider or healthcare worker or home health aides or personnel, health or public health officer).mp. [mp=title, abstract, heading word, table of contents, key concepts, original title, tests & measures, mesh]

EMBASE:

('health care personnel'/exp OR 'health care personnel' OR 'health care practitioner' OR 'health care professional' OR 'health care provider' OR 'health care worker' OR 'health personnel' OR 'health profession personnel' OR 'health worker' OR 'healthcare personnel' OR 'healthcare practitioner' OR

'healthcare professional' OR 'healthcare provider' OR 'healthcare worker' OR 'home health aides' OR 'personnel, health' OR 'public health officer') AND ('sars coronavirus'/exp OR 'hcov-sars' OR 'human sars coronavirus' OR 'sars cov' OR 'sars associated coronavirus' OR 'sars coronavirus' OR 'sars virus' OR 'sars-cov' OR 'sars-associated coronavirus' OR 'severe acute respiratory syndrome coronavirus' OR 'severe acute respiratory syndrome virus' OR 'middle east respiratory syndrome coronavirus'/exp OR 'mers coronavirus' OR 'mers virus' OR 'mers-cov' OR 'middle east respiratory syndrome coronavirus' OR 'covid 19'/exp) AND ('stress'/exp OR 'alarm reaction' OR 'stress' OR 'stress capacity' OR 'stress reaction' OR 'stress resistance' OR 'stress response' OR 'stress situation' OR 'stress tolerance' OR 'relaxation training'/exp OR 'mind-body and relaxation techniques' OR 'relaxation method' OR 'relaxation technics' OR 'relaxation techniques' OR 'relaxation therapy' OR 'relaxation training' OR 'training, relaxation' OR 'breathing exercise'/exp OR 'breathing exercise' OR 'breathing exercises' OR 'breathing therapy' OR 'chest physical therapy' OR 'chest physiotherapy' OR 'exercise, breathing' OR 'exercise, respiratory' OR 'respiration exercise' OR 'respiration therapy' OR 'respiratory exercise' OR 'respiratory physiotherapy' OR 'psychology'/exp OR 'cognitive science' OR 'psychology' OR 'psychology, applied' OR 'psychology, comparative' OR 'psychology, educational' OR 'psychology, experimental' OR 'psychology, industrial' OR 'psychology, military' OR 'schizophrenic psychology' OR 'mindfulness'/exp OR 'mindfulness' OR 'alternative medicine'/exp OR 'alternative medicine' OR 'alternative therapies' OR 'alternative therapy' OR 'complementary medicine' OR 'complementary therapies' OR 'diet fads' OR 'eclecticism, historical' OR 'mental healing' OR 'mind body technique' OR 'mind body therapies' OR 'mind body therapy' OR 'mind-body relations (metaphysics)' OR 'mind-body relations, metaphysical' OR 'mind-body therapies' OR 'polarity therapy' OR 'radiaesthesia' OR 'radiesthesia' OR 'reflexotherapy' OR 'therapeutic cults' OR 'meditation'/exp OR 'meditation' OR 'psychotherapy'/exp OR 'holistic psychotherapy' OR 'psychotherapeutic processes' OR 'psychotherapeutic training' OR 'psychotherapy' OR 'psychotherapy, multiple' OR 'socioenvironmental therapy' OR 'mental health'/exp OR 'condition, mental' OR 'health, mental' OR 'mental care' OR 'mental condition' OR 'mental factor' OR 'mental health' OR 'mental help' OR 'mental service' OR 'mental state' OR 'mental status' OR 'mental status schedule' OR 'psychic health' OR 'anxiety'/exp OR 'anxiety' OR 'depression psychology'/exp OR 'insomnia'/exp OR 'agrypnia' OR 'hyposomnia' OR 'insomnia' OR 'sleep initiation and maintenance disorders' OR 'sleeplessness' OR 'posttraumatic stress disorder'/exp OR 'ptsd' OR 'combat disorders' OR 'combat fatigue' OR 'combat stress' OR 'post-traumatic stress' OR 'post-traumatic stress disorder' OR 'posttraumatic neurosis' OR 'posttraumatic psychic syndrome' OR 'posttraumatic psychosis' OR 'posttraumatic stress' OR 'posttraumatic stress disorder' OR 'posttraumatic syndrome' OR 'psychosis, posttraumatic' OR 'stress disorders, post-traumatic' OR 'stress disorders, traumatic' OR 'stress, posttraumatic' OR 'trauma and stressor related disorders' OR 'traumatic stress' OR 'traumatic stress disorder' OR 'war neurosis' OR 'burnout'/exp OR 'burn-out' OR 'burn-out syndrome' OR 'burnout' OR 'burnout syndrome' OR 'burnout, psychological' OR 'psychological burn-out' OR 'psychological burnout' OR 'stigma'/exp OR 'stigma' OR 'quality of life'/exp OR 'hrql' OR 'health related quality of life' OR 'life quality' OR 'quality of life')

CINAHL

(MH "Relaxation Techniques") OR ((MH "Stress") OR (MH "Stress Disorders, Post-Traumatic") OR (MH "Stress, Psychological")) OR ('stress' OR 'alarm reaction' OR 'stress capacity' OR 'stress reaction' OR 'stress resistance' OR 'stress response' OR 'stress situation' OR 'stress tolerance' OR 'relaxation training' OR 'mind-body and relaxation techniques' OR 'relaxation method' OR 'relaxation

technics' OR 'relaxation techniques' OR 'relaxation therapy' OR 'relaxation training' OR 'training, relaxation' OR 'breathing exercise' OR 'breathing exercises' OR 'breathing therapy' OR 'chest physical therapy' OR 'chest physiotherapy' OR 'exercise, breathing' OR 'exercise, respiratory' OR 'respiration exercise' OR 'respiration therapy' OR 'respiratory exercise' OR 'respiratory physiotherapy' OR 'psychology' OR 'cognitive science' OR 'psychology' OR 'psychology, applied' OR 'psychology, comparative' OR 'psychology, educational' OR 'psychology, experimental' OR 'psychology, industrial' OR 'psychology, military' OR 'schizophrenic psychology' OR 'mindfulness'/exp OR 'mindfulness' OR 'alternative medicine' OR 'alternative medicine' OR 'alternative therapies' OR 'alternative therapy' OR 'complementary medicine' OR 'complementary therapies' OR 'diet fads' OR 'eclecticism, historical' OR 'mental healing' OR 'mind body technique' OR 'mind body therapies' OR 'mind body therapy' OR 'mind-body relations (metaphysics)' OR 'mind-body relations, metaphysical' OR 'mind-body therapies' OR 'polarity therapy' OR 'radiaesthesia' OR 'radiesthesia' OR 'reflexotherapy' OR 'therapeutic cults' OR 'meditation' OR 'meditation' OR 'psychotherapy' OR 'holistic psychotherapy' OR 'psychotherapeutic processes' OR 'psychotherapeutic training' OR 'psychotherapy' OR 'psychotherapy, multiple' OR 'socioenvironmental therapy' OR 'mental health' OR 'condition, mental' OR 'health, mental' OR 'mental care' OR 'mental condition' OR 'mental factor' OR 'mental health' OR 'mental help' OR 'mental service' OR 'mental state' OR 'mental status' OR 'mental status schedule' OR 'psychic health' OR 'anxiety' OR 'anxiety' OR 'depression psychology' OR 'insomnia' OR 'agrypnia' OR 'hyposomnia' OR 'insomnia' OR 'sleep initiation and maintenance disorders' OR 'sleeplessness' OR 'posttraumatic stress disorder' OR 'ptsd' OR 'ptsd (posttraumatic stress disorder)' OR 'combat disorders' OR 'combat fatigue' OR 'combat stress' OR 'post-traumatic stress' OR 'post-traumatic stress disorder' OR 'posttraumatic neurosis' OR 'posttraumatic psychic syndrome' OR 'posttraumatic psychosis' OR 'posttraumatic stress' OR 'posttraumatic stress disorder' OR 'posttraumatic syndrome' OR 'psychosis, posttraumatic' OR 'stress disorders, post-traumatic' OR 'stress disorders, traumatic' OR 'stress, posttraumatic' OR 'trauma and stressor related disorders' OR 'traumatic stress' OR 'traumatic stress disorder' OR 'war neurosis' OR 'burnout' OR 'burn-out' OR 'burn-out syndrome' OR 'burnout syndrome' OR 'burnout, psychological' OR 'psychological burn-out' OR 'psychological burnout' OR 'stigma' OR 'quality of life' OR 'hrql' OR 'health related quality of life' OR 'life quality' OR 'quality of life' AND ((MH "Coronavirus") OR (MH "Coronavirus Infections") OR (MH "Middle East Respiratory Syndrome Coronavirus") OR (MH "SARS Virus") OR (MH "Middle East Respiratory Syndrome")) OR ('sars coronavirus' OR 'hcov-sars' OR 'human sars coronavirus' OR 'sars cov' OR 'sars associated coronavirus' OR 'sars coronavirus' OR 'sars virus' OR 'sars-cov' OR 'sars-associated coronavirus' OR 'severe acute respiratory syndrome coronavirus' OR 'severe acute respiratory syndrome virus' OR 'middle east respiratory syndrome coronavirus' OR 'mers coronavirus' OR 'mers virus' OR 'mers-cov' OR 'middle east respiratory syndrome coronavirus' OR 'covid 19') AND ((MH "Community Health Workers") OR (MH "Health Personnel")) OR ('health care personnel' OR 'health care practitioner' OR 'health care professional' OR 'health care provider' OR 'health care worker' OR 'health personnel' OR 'health profession personnel' OR 'health worker' OR 'healthcare personnel' OR 'healthcare practitioner' OR 'healthcare professional' OR 'healthcare provider' OR 'healthcare worker' OR 'home health aides' OR 'personnel, health' OR 'public health officer')

GOOGLE SCHOLAR

(Covid-19 OR Sars OR Mers AND health care workers AND mindfulness)
